# Pharmacovigilance Analysis of Bleeding Events Associated With Selective Serotonin Reuptake Inhibitors (SSRIs) Using the US Food and Drug Administration Adverse Event Reporting System (FAERS)

**DOI:** 10.7759/cureus.110163

**Published:** 2026-06-03

**Authors:** Adrian Chin Yan Chan

**Affiliations:** 1 Pharmacology, Bayer Pharmaceuticals, Beijing, CHN

**Keywords:** bleeding risk, drug safety, faers, hemorrhagic events, pharmacovigilance, selective serotonin reuptake inhibitors

## Abstract

Background

Selective serotonin reuptake inhibitors (SSRIs) are commonly prescribed for depressive and anxiety disorders and may increase the risk of bleeding. However, comprehensive real-world data on the patterns and distribution of SSRI-related bleeding remain limited. This study aimed to characterize bleeding adverse events associated with individual SSRIs using large-scale pharmacovigilance data.

Methods

A retrospective, descriptive pharmacovigilance study was conducted using data from the US Food and Drug Administration Adverse Event Reporting System (FAERS), retrieved from the first quarter of 2004 to the fourth quarter of 2025. Reports containing sertraline, citalopram, escitalopram, fluoxetine, paroxetine, or fluvoxamine as suspect drugs were identified and filtered using the Medical Dictionary for Regulatory Activities (MedDRA) System Organ Class (SOC) “Vascular disorders” and the High-Level Group Term (HLGT) “Vascular hemorrhagic disorders NEC”. Bleeding events were summarized at the Preferred Term (PT) level. Descriptive analyses were performed for demographic characteristics, geographic distribution, bleeding sites, and concomitant use of medications associated with bleeding risk.

Results

A total of 5,604 bleeding-related cases were identified across 380,241 total FAERS cases. Bleeding event rates varied by SSRI: escitalopram (2.85%), citalopram (1.93%), paroxetine (1.29%), sertraline (1.25%), fluvoxamine (1.05%), and fluoxetine (1.02%). Sertraline accounted for the highest absolute number of bleeding reports (1,352, 24.1%), followed by escitalopram (1,229, 21.9%) and citalopram (1,029, 18.4%). Among the cases with available age data, patients aged >65 years constituted the largest group (range: 17.1%-46.6%), followed by adults aged 18-65 years (range: 11.0%-24.0%). Female patients were predominant (2,926, 52.2%). Hemorrhage (unspecified) was the most frequently reported event (range: 5.99%-15.93%), followed by contusion (0.70%-10.24%) and hematoma (1.25%-4.52%). Gastrointestinal (GI) bleeding events, including GI hemorrhage (0.37%-1.45%) and rectal hemorrhage (0.31%-5.94%), and central nervous system bleeding, including cerebral hemorrhage (0.40%-1.27%) and subarachnoid hemorrhage (0.35%-1.16%), were consistently reported. Concomitant use of medications increasing bleeding risk was documented in 541 cases (9.7%), with antiplatelet agents being most frequent (246, 4.4%), followed by nonsteroidal anti-inflammatory drugs (NSAIDs) (143, 2.6%), direct oral anticoagulants (DOACs) (130, 2.3%), and warfarin (22, 0.4%).

Conclusion

This FAERS-based analysis demonstrated that bleeding represented a class-wide safety concern among SSRIs. There were differences in bleeding patterns within the FAERS data; however, these findings are descriptive and should not be interpreted as comparative risk differences. These findings underscore the importance of individualized risk assessment, careful medication reconciliation, and strengthened pharmacovigilance to support safer SSRI prescription in routine clinical practice.

## Introduction

Selective serotonin reuptake inhibitors (SSRIs) are among the most commonly prescribed antidepressants worldwide. They are widely used for the management of depression, anxiety disorders, premenstrual dysphoric disorder, chronic pain syndromes, and vasomotor symptoms [1,2]. Their wide-ranging signs and protracted use in various groups of patients have attracted growing concern about the problem of adverse drug reactions, especially complications of bleeding. Despite the general perception that SSRIs are safe, a growing body of evidence demonstrates that they can affect hemostasis and make people susceptible to hemorrhagic events, which is clinically significant and could be prevented [3].

The association between SSRIs and bleeding risk is primarily attributed to their effects on platelet function and gastrointestinal (GI) physiology. Platelets are unable to produce serotonin but instead take it through absorption into the plasma through the serotonin transporter (SERT). When platelets are injured, serotonin stored within them is released, activating platelet 5-hydroxytryptamine receptor 2A (5-HT2A) receptors to promote platelet aggregation and clotting [4,5]. By inhibiting SERT, SSRIs reduce intraplatelet serotonin levels, thereby impairing platelet aggregation and weakening primary hemostasis [4-6].

A second proposed mechanism involves SSRI-mediated effects on gastric acid secretion. Serotonin plays a regulatory role in GI physiology, and vagal stimulation triggers serotonin release in the GI tract, where serotonin receptors modulate gastric secretory activity. SSRIs are believed to increase basal gastric acid secretion by enhancing serotonergic signaling, which may predispose patients to mucosal injury, ulcer formation, and GI bleeding [7]. Together, platelet dysfunction and increased gastric acidity represent complementary biological pathways through which SSRIs may elevate bleeding risk.

In addition to these mechanisms, pharmacokinetic drug-drug interactions mediated by cytochrome (CYP) P450 enzymes further contribute to bleeding risk. Certain SSRIs are potent inhibitors of CYP isoenzymes and can increase plasma concentrations of anticoagulant or antiplatelet medications. Fluvoxamine (strong CYP1A2, 2C9, 2C19 inhibition), fluoxetine (strong CYP2D6 inhibition), and paroxetine (strong CYP2D6 inhibition) are the most clinically relevant CYP inhibitors among SSRIs [8,9]. In contrast, citalopram, escitalopram, and sertraline exhibit minimal CYP inhibition and are generally considered to have a lower interaction-related bleeding risk [9]. However, the current FAERS-based analysis was descriptive in nature and was not designed to formally evaluate drug-drug interaction effects or establish causal relationships. Therefore, the discussion of CYP-mediated mechanisms should be interpreted as biologically plausible and hypothesis-generating based on existing literature [8,9]

Several systematic reviews and meta-analyses have demonstrated a significant association between SSRI use and bleeding outcomes. Laporte et al. conducted a large meta-analysis of observational studies and reported a significantly increased risk of overall bleeding among SSRI users, with the strongest association observed for GI hemorrhage [10]. Bixby and colleagues synthesized clinical data demonstrating that SSRI-associated bleeding has been documented across multiple anatomical sites, particularly in patients without individualized bleeding risk assessment or with concomitant antithrombotic therapy [3]. More recently, Edinoff et al. integrated mechanistic and clinical evidence, concluding that SSRIs are associated with abnormal bleeding events and emphasizing the need for heightened pharmacovigilance, especially in high-risk populations such as older adults, patients with a history of GI ulcer or prior bleeding, individuals with hepatic impairment, and those receiving concomitant antiplatelet agents, anticoagulants, or nonsteroidal anti-inflammatory drugs (NSAIDs) [11].

Clinically reported SSRI-associated bleeding events occur across a wide range of anatomical sites. The most frequently reported site is the GI tract, while intracranial hemorrhage, although rare, represents a serious and potentially fatal complication [10,11]. The risk of bleeding may be further increased when SSRIs are used concomitantly with other medications known to impair hemostasis, including NSAIDs, aspirin, anticoagulants, antiplatelet agents, corticosteroids, and serotonergic drugs such as tramadol. Common drug classes known to increase bleeding risk include antiplatelet agents (such as aspirin and clopidogrel), anticoagulants (including warfarin and direct oral anticoagulants (DOACs)), NSAIDs, and corticosteroids. Other reported bleeding manifestations include epistaxis, gingival bleeding, subconjunctival hemorrhage, vaginal and gynecological bleeding, epidural hematoma, hemorrhagic patellar bursitis, retrobulbar hematoma, and bleeding into joints [3,11]. The risk of bleeding is further amplified when SSRIs are used concomitantly with medications known to impair hemostasis. In addition to antiplatelet agents, anticoagulants, and NSAIDs, other serotonergic drug classes may also contribute to hemorrhagic complications when co-administered with SSRIs. Tramadol, a centrally acting analgesic with serotonin reuptake inhibitory activity, has been associated with serotonin syndrome and related vascular adverse effects. Excess serotonergic activity may alter platelet aggregation, vascular tone, and endothelial function, thereby potentially increasing susceptibility to bleeding events. Previous reports have highlighted clinically important interactions between SSRIs and serotonergic agents, emphasizing the need for cautious concomitant use and monitoring for hemorrhagic complications and serotonin toxicity [12,13]. According to Bixby et al., aspirin, clopidogrel, warfarin, and NSAIDs are supported by robust clinical evidence as agents that significantly increase the risk of SSRI-associated bleeding when used concurrently [3].

Despite substantial evidence linking SSRIs to bleeding risk, critical gaps remain in the characterization of these adverse events. Regulatory documents such as the UK Summary of Product Characteristics (SPCs) for commonly prescribed SSRIs acknowledge hemorrhagic adverse reactions but lack detailed information regarding bleeding site specificity, frequency, severity, and patient characteristics [14]. For example, the fluvoxamine SPC broadly lists GI, gynecological, mucocutaneous, and postpartum hemorrhage without granular classification or quantitative risk estimates [14]. Consequently, the true distribution and anatomical patterns of SSRI-associated bleeding events remain incompletely characterized in the existing literature, which has largely focused on overall bleeding risk rather than detailed site-specific phenotyping [3,10,11]. 

Spontaneous adverse event reporting systems provide a valuable opportunity to investigate rare, severe, and real-world drug-related bleeding events at a population level. The US Food and Drug Administration Adverse Event Reporting System (FAERS) captures safety data across diverse clinical settings and patient populations, enabling systematic evaluation of hemorrhagic adverse events that may not be fully identified during pre-approval clinical trials [15,16]. A cumulative and comparative analysis of individual SSRIs may help clarify both class-wide and drug-specific bleeding patterns.

The primary aim of this study was to descriptively characterize hemorrhagic adverse events associated with six commonly prescribed SSRIs using FAERS data. The secondary objectives were to describe demographic patterns of reported cases, identify frequently reported bleeding sites using Medical Dictionary for Regulatory Activities (MedDRA) terminology, and assess the presence of concomitant medications associated with bleeding risk. This study is exploratory and descriptive in nature and does not aim to estimate incidence, relative risk, or establish causality between individual SSRIs and bleeding outcomes. To support these objectives, a structured MedDRA-based classification approach was used to systematically identify hemorrhagic events and enable consistent descriptive analysis across SSRIs.

## Materials and methods

This study aimed to perform a cumulative review and comprehensive characterization of hemorrhagic adverse events associated with six commonly prescribed SSRIs, such as sertraline, citalopram, escitalopram, fluoxetine, paroxetine, and fluvoxamine, using safety data from the FAERS.

Study design

This was a retrospective, descriptive pharmacovigilance study based on spontaneous adverse event reports. A cumulative analysis was conducted for each SSRI using FAERS data retrieved from the first quarter (Q1) of 2004 to the fourth quarter (Q4) of 2025. The study design was observational and non-interventional, intended to describe patterns and characteristics of reported hemorrhagic adverse events rather than to establish causality or estimate incidence rates. This analysis was purely descriptive and exploratory. No comparative safety evaluation, signal detection analysis, or causal inference was performed.

Data source

Data were retrieved from the publicly accessible FDA FAERS Dashboard for the period from Q1 of 2004 to Q4 of 2025 [16]. FAERS is a publicly available pharmacovigilance database designed to support post-marketing drug safety surveillance. Only dashboard-based extraction was performed for this study; quarterly ASCII/AEMS raw data files were neither downloaded nor processed. The database contains adverse event reports submitted by healthcare professionals, consumers, and manufacturers. This study utilized aggregated dashboard-derived data for the descriptive characterization of bleeding-related adverse event reporting patterns. As only publicly available de-identified aggregated data were used, no formal data request, patient consent, or ethics approval was required.

Study population and case identification

Data Cleaning and Case Deduplication

For each of the six SSRIs, all FAERS cases reporting the respective drug as a primary suspect (PS) or secondary suspect (SS) product were initially identified. Concomitant (C) and interacting (I) roles were not used for primary case inclusion. However, other co-administered medications known to increase bleeding risk (e.g., NSAIDs, antiplatelet agents, and anticoagulants) were identified separately and analyzed as concomitant exposures. To ensure comprehensive capture of reports, all known generic, branded, and salt formulations corresponding to each SSRI were included in the case identification process. From this initial cohort, cases were subsequently screened for the presence of at least one adverse event coded under the MedDRA System Organ Class (SOC) “Vascular disorders”. FAERS data were cleaned and deduplicated according to FDA recommendations. When multiple reports corresponded to the same CASEID, only the most recent version (based on FDA_DT) was retained. If duplicate CASEIDs appeared across reporting quarters, the latest case version was preserved, and earlier submissions were excluded. Records with missing CASEID or PS drug information were excluded prior to analysis. The complete list of active ingredient names used for SSRI identification in FAERS is provided in Table 1.

**Table 1:** Active ingredient names and common brand names used for SSRI identification in FAERS Brand names were included in the DRUG file search strategy to ensure comprehensive capture of reports across international submissions. SSRI: selective serotonin reuptake inhibitors, FAERS: FDA Adverse Event Reporting System.

SSRI drug	Active ingredient names
Sertraline	Sertraline hydrochloride
Citalopram	Citalopram hydrobromide or hydrochloride
Paroxetine	Paroxetine hydrochloride or hydrochloride hemihydrate or mesylate
Fluoxetine	Fluoxetine hydrochloride
Fluvoxamine	Fluvoxamine maleate
Escitalopram	Escitalopram oxalate

No restriction was applied based on indication for SSRI use; cases were included regardless of reported therapeutic indication (e.g., depression, anxiety disorders, vasomotor symptoms, premenstrual dysphoric disorder, chronic pain syndromes, or other off-label uses), reflecting real-world prescribing patterns. Cases were not filtered or selected based on prior literature reports, comorbidities, or clinical severity. Inclusion was based solely on FAERS database criteria and MedDRA coding.

No age or sex restrictions were applied during case inclusion. All age groups (<18 years, 18-65 years, >65 years) and both sexes were included. Cases with missing demographic information were retained and analyzed descriptively. Subsequently, cases were further refined to include only those reporting at least one adverse event within the MedDRA High-Level Group Term (HLGT) “Vascular hemorrhagic disorders NEC”. This HLGT was selected as it captures the majority of clinically relevant hemorrhagic events. No additional Preferred Terms (PT) outside this hierarchy were included to maintain methodological simplicity. This approach ensured comprehensive capture of hemorrhagic adverse events while excluding non-hemorrhagic vascular conditions. Cases that did not meet these hemorrhagic criteria were excluded from the final analytical dataset.

Each included case could report multiple adverse events and multiple occurrences of the same event; therefore, the total number of reported events exceeded the total number of cases.

Adverse Event Classification

Adverse events were classified using the MedDRA®, a standardized international medical terminology developed by the International Council for Harmonization (ICH) [17]. MedDRA terminology was accessed through the publicly available FAERS database interface. No separate license or additional permission was required for its use in this study. No additional standardization was performed to harmonize MedDRA versions across the study period, as no significant impact on descriptive findings was expected.

**Figure 1:**
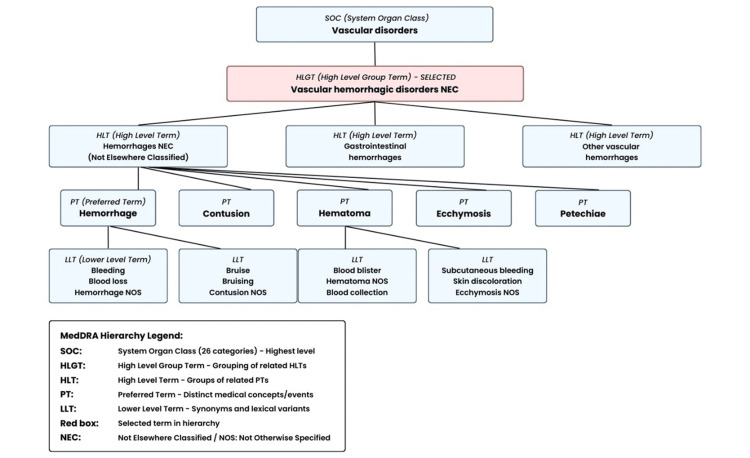
MedDRA hierarchy diagram Concomitant medications associated with bleeding risk [18]. MedDRA: Medical Dictionary for Regulatory Activities. Image credit: Created by the author using Google Docs (Google Workspace, Google, Mountain View, CA, USA).

This hierarchical approach, illustrated in Figure 1, enabled the standardized and reproducible identification of hemorrhagic adverse events across all SSRIs.

Within the final dataset, reports indicating concomitant or suspect use of commonly prescribed oral medications known to increase bleeding risk, based on established clinical literature and prior pharmacovigilance studies [3,7,10], were identified. These medications were classified into four predefined pharmacological groups: NSAIDs, antiplatelet agents, warfarin, and DOACs. These drug classes are known to increase bleeding risk through mechanisms such as impaired platelet aggregation (NSAIDs and antiplatelet agents), inhibition of coagulation pathways (warfarin and DOACs), and increased GI mucosal vulnerability (NSAIDs). Each case was counted once per medication group, and instances of exposure to multiple bleeding-risk medications were documented where applicable (Table 2).

**Table 2:** Commonly prescribed oral medications that are known to be associated with an increased risk of bleeding NSAIDs: nonsteroidal anti-inflammatory drugs, DOACs: direct oral anticoagulants.

Group A (NSAIDs)	Group B (antiplatelets)	Group C (Warfarin)	Group D (DOACs)
Ketorolac	Aspirin	Warfarin	Rivaroxaban
Piroxicam	Clopidogrel		Apixaban
Meloxicam	Ticagrelor		Edoxaban
Indomethacin	Prasugrel		Dabigatran
Ketoprofen	Cilostazol		
Naproxen	Dipyridamole		
Diclofenac			
Ibuprofen			

Statistical analysis

Descriptive statistical analyses were conducted to characterize demographic variables, geographic distribution, hemorrhagic adverse event profiles, and concomitant medication use. Categorical variables were summarized using frequencies and percentages. Percentages for adverse events were calculated using the total number of cases for each SSRI as the denominator.

All analyses were performed using Statistical Analysis System (SAS) software, version 9.4 (SAS Institute Inc., Cary, NC, USA) [19]. No inferential statistical testing or signal disproportionality analyses were conducted, consistent with the study's descriptive objectives. Within each case, repeated occurrences of the same PT were counted only once to avoid duplication.

Ethical considerations

This study used publicly available, de-identified FAERS data with no access to individual patient identifiers. In accordance with regulatory guidance and institutional policies, ethical approval and informed consent were not required for this research.

## Results

**Figure 2:**
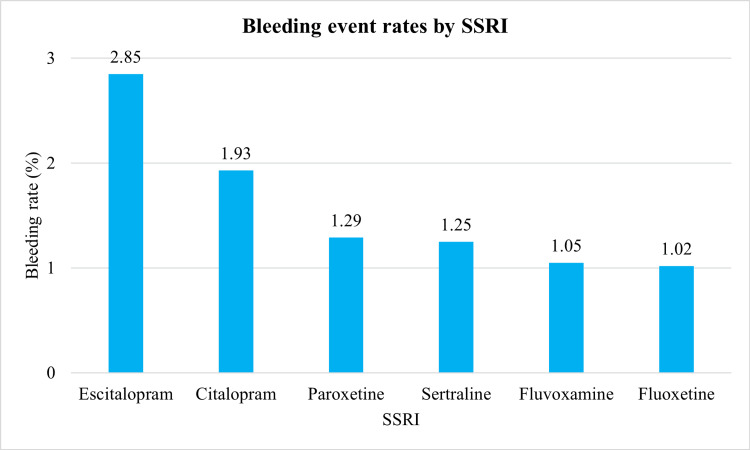
Case distribution by SSRI SSRI: serotonin reuptake inhibitor.

A total of 5,604 cases reporting bleeding-related adverse events associated with SSRIs were identified in the FAERS database. The bleeding event rates across individual SSRIs are given in Table 3 and Figure 2. Bleeding event rates varied by SSRI: escitalopram (1,229, 2.85%), citalopram (1,029, 1.93%), paroxetine (940, 1.29%), sertraline (1,352, 1.25%), fluvoxamine (82, 1.05%), and fluoxetine (972, 1.02%). Escitalopram demonstrated the highest proportion of bleeding reports (1,229, 2.85%), followed by citalopram (1,029, 1.93%), while fluoxetine showed the lowest proportion (972, 1.02%).

**Table 3:** Bleeding event rates by SSRI SSRI: selective serotonin reuptake inhibitor; FAERS: FDA Adverse Event Reporting System.

SSRI	Bleeding cases	Total FAERS cases	Bleeding rate (%)
Sertraline	1,352	108,307	1.25
Citalopram	1,029	53,185	1.93
Paroxetine	940	72,992	1.29
Fluoxetine	972	94,844	1.02
Fluvoxamine	82	7,816	1.05
Escitalopram	1,229	43,097	2.85

**Figure 3:**
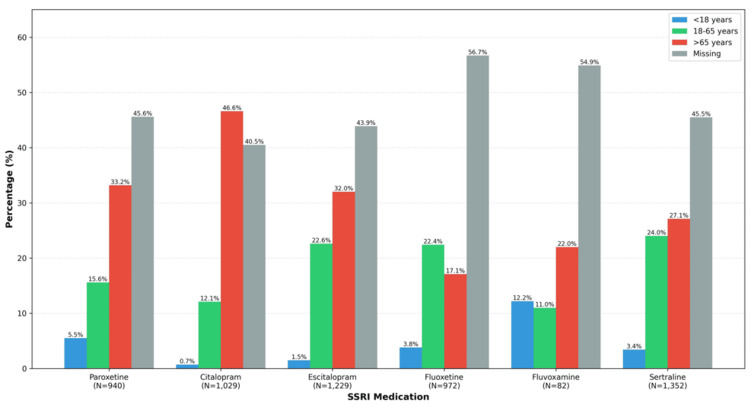
Age distribution across SSRIs SSRI: serotonin reuptake inhibitor.

Among cases with available age information, patients aged >65 years constituted the largest group across all SSRIs (range: 17.1%-46.6%), followed by adults aged 18-65 years (range: 11.0%-24.0%) and pediatric patients aged <18 years (range: 0.7%-12.2%). A substantial proportion of cases had missing age data, ranging from 417 cases (40.5%) (citalopram) to 551 cases (56.7%) (fluoxetine), limiting robust age-stratified comparisons across drugs (Table 4, Figure 3). Across all SSRIs, female patients predominated, accounting for 2,926 cases (52.2%), compared with 2,141 male patients (38.2%). Gender was not specified in 537 cases (9.6%). Female predominance was observed consistently across individual SSRIs (except for escitalopram), ranging from 629 cases (46.5%) (sertraline) to 58 cases (70.7%) (fluvoxamine) (Table 4).

**Table 4:** Age and gender distribution across SSRIs SSRI: selective serotonin reuptake inhibitor.

	Paroxetine (N = 940)	Citalopram (N = 1,029)	Escitalopram (N = 1,229)	Fluoxetine (N = 972)	Fluvoxamine (N = 82)	Sertraline (N = 1,352)
Age category						
<18 years	52 (5.5%)	7 (0.7%)	18 (1.5%)	37 (3.8%)	10 (12.2%)	46 (3.4%)
18-65 years	147 (15.6%)	125 (12.1%)	278 (22.6%)	218 (22.4%)	9 (11.0%)	325 (24.0%)
>65 years	312 (33.2%)	480 (46.6%)	393 (32.0%)	166 (17.1%)	18 (22.0%)	366 (27.1%)
Missing	429 (45.6%)	417 (40.5%)	540 (43.9%)	551 (56.7%)	45 (54.9%)	615 (45.5%)
Gender						
Female	601 (63.9%)	561 (54.5%)	541 (44.0%)	536 (55.1%)	58 (70.7%)	629 (46.5%)
Male	269 (28.6%)	390 (37.9%)	547 (44.5%)	345 (35.5%)	24 (29.3%)	566 (41.9%)
Not specified	70 (7.4%)	78 (7.6%)	141 (11.5%)	91 (9.4%)	0	157 (11.6%)

**Figure 4:**
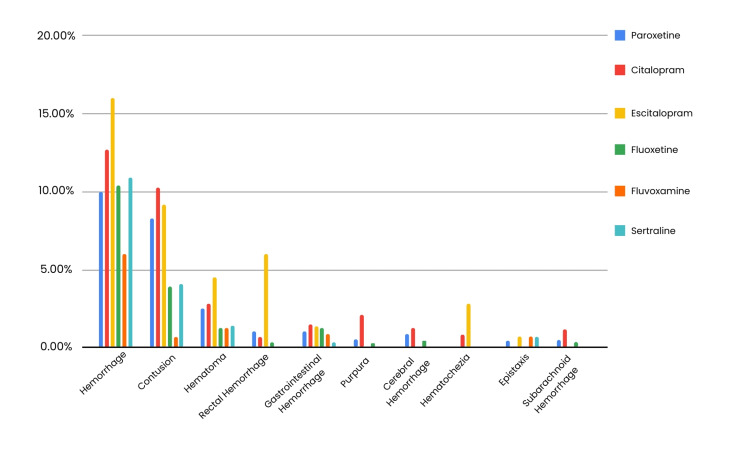
Distribution of top bleeding-related MedDRA Preferred Terms across SSRIs MedDRA: Medical Dictionary for Regulatory Activities; SSRI: serotonin reuptake inhibitor.

Across all SSRIs, hemorrhage (unspecified) was the most frequently reported MedDRA PT, ranging from 5.99% to 15.93% of total FAERS cases, followed by contusion (0.70% to 10.24%) and hematoma (1.25% to 4.52%). GI bleeding events, including GI hemorrhage (0.37%-1.45%), rectal hemorrhage (0.31%-5.94%), and hematochezia (0.76%-2.84%), were consistently reported across SSRIs, with GI hemorrhage observed in all six drugs. Central nervous system (CNS) hemorrhagic events, such as cerebral hemorrhage (0.40%-1.27%) and subarachnoid hemorrhage (0.35%-1.16%), were reported less frequently and were observed only for paroxetine, citalopram, and fluoxetine. Notable drug-specific bleeding patterns emerged from the analysis. Escitalopram demonstrated the highest rates of hemorrhage (15.93%), rectal hemorrhage (5.95%), and hematochezia (2.84%) among all SSRIs. Fluvoxamine showed distinct findings with only five bleeding event types appearing in the top 10 most frequently reported events: hemorrhage (5.99%), contusion (0.70%), hematoma (1.28%), GI hemorrhage (0.84%), and epistaxis (0.56%). Notably, fluvoxamine did not report rectal hemorrhage, purpura, cerebral hemorrhage, hematochezia, or subarachnoid hemorrhage among its top 10 bleeding events, and showed substantially lower frequencies for hemorrhage and contusion compared to other SSRIs in the dataset (Table 5, Figure 4).

**Table 5:** Most frequently reported bleeding-related MedDRA PT across SSRIs Events shown represent the most frequently reported bleeding terms across all SSRIs. Percentages are calculated as the proportion of total FAERS cases for each drug reporting each specific event. Dashes (-) indicate no cases reported or the event was not in the top 10 for that specific SSRI. GI: gastrointestinal, MedDRA: Medical Dictionary for Regulatory Activities, PT: preferred term, SSRI: selective serotonin reuptake inhibitors.

PT	Paroxetine (N = 72,992)	Citalopram (N = 53,185)	Escitalopram (N = 43,097)	Fluoxetine (N = 94,844)	Fluvoxamine (N = 7,816)	Sertraline (N = 108,307)
	n (%)					
Hemorrhage	7,254 (9.94%)	6,747 (12.69%)	6,864 (15.93%)	9,828 (10.36%)	468 (5.99%)	11,778 (10.87%)
Contusion	5,995 (8.21%)	5,445 (10.24%)	3,960 (9.19%)	3,740 (3.94%)	55 (0.70%)	4,400 (4.06%)
Hematoma	1,840 (2.52%)	1,470 (2.76%)	1,950 (4.52%)	1,190 (1.25%)	100 (1.28%)	1,510 (1.39%)
Rectal hemorrhage	735 (1.01%)	357 (0.67%)	2,562 (5.94%)	294 (0.31%)	-	-
GI hemorrhage	462 (0.63%)	770 (1.45%)	594 (1.38%)	1,210 (1.28%)	66 (0.84%)	396 (0.37%)
Purpura	399 (0.55%)	1,121 (2.11%)	-	285 (0.30%)	-	-
Cerebral hemorrhage	650 (0.89%)	675 (1.27%)	-	375 (0.40%)	-	-
Hematochezia	-	405 (0.76%)	1,224 (2.84%)	-	-	-
Epistaxis	286 (0.39%)	-	308 (0.71%)	-	44 (0.56%)	748 (0.69%)
Subarachnoid hemorrhage	336 (0.46%)	616 (1.16%)	-	336 (0.35%)	-	-

A total of 541 cases (9.7%) reported concomitant use of at least one medication known to increase bleeding risk. Antiplatelet agents were the most frequently reported (246, 4.4%), followed by NSAIDs (143, 2.6%), DOACs (130, 2.3%), and warfarin (22, 0.4%). The proportion of cases with concomitant bleeding-risk medications varied across SSRIs, ranging from 3.7% (fluvoxamine) to 11.5% (citalopram). The vast majority of cases reported only one such medication, with very rare reports of multiple agents (Table 6).

**Table 6:** Concomitant use of oral medications associated with bleeding risk *Two cases (one citalopram, one fluoxetine) reported concomitant use of two medications associated with bleeding risk; all other cases reported only one such medication. DOACs: direct oral anticoagulants, NSAIDs: nonsteroidal anti-inflammatory drugs.

Drug	NSAIDs	Antiplatelets	Warfarin	DOACs	Any bleeding-risk medication
Paroxetine	6 (0.64%)	30 (3.19%)	3 (0.32%)	11 (1.17%)	50 (5.32%)
Citalopram	34 (3.30%)	71 (6.90%)	5 (0.49%)	8 (0.78%)	118 (11.47%)
Escitalopram	25 (2.03%)	64 (5.21%)	7 (0.57%)	44 (3.58%)	140 (11.39%)
Fluoxetine	22 (2.26%)	37 (3.81%)	6 (0.62%)	22 (2.26%)	87 (8.95%)
Fluvoxamine	0	3 (3.66%)	0	0	3 (3.66%)
Sertraline	56 (4.14%)	41 (3.03%)	1 (0.07%)	45 (3.33%)	143 (10.58%)*

Bleeding event profiles for each SSRI

Among 72,992 paroxetine-associated bleeding cases, the most frequently reported events were hemorrhage (unspecified) (9.94%), contusion (8.21%), hematoma (2.52%), and rectal hemorrhage (1.01%). CNS hemorrhages, including cerebral hemorrhage (0.89%) and subarachnoid hemorrhage (0.46%), and GI bleeding events (0.63%) represented clinically significant findings.

In 53,185 citalopram cases, hemorrhage (unspecified) (6,747, 12.69%), contusion (5,445, 10.24%), hematoma (1,470, 2.76%), and purpura (1,121, 2.11%) predominated. A broad spectrum of GI bleeding events was notable, including GI hemorrhage (770, 1.45%), hematochezia (405, 0.76%), hematemesis (385, 0.72%), and rectal hemorrhage (357, 0.67%), alongside frequent CNS hemorrhagic reports including cerebral hemorrhage (675, 1.27%) and subarachnoid hemorrhage (616, 1.16%).

Among 43,097 escitalopram cases, hemorrhage (unspecified) (6,864, 15.93%), contusion (3,960, 9.19%), rectal hemorrhage (2,562, 5.94%), and hematoma (1,950, 4.52%) were most frequently reported. Rectal hemorrhage occurred at a markedly higher frequency compared with other SSRIs. Additional GI events included hematochezia (1,224, 2.84%), GI hemorrhage (594, 1.38%), and hematemesis (363, 0.84%). Respiratory tract bleeding events, including hemoptysis (535, 1.24%) and pulmonary alveolar hemorrhage (495, 1.15%), were also prominent.

In 94,844 fluoxetine cases, hemorrhage (unspecified) (9,828, 10.36%), contusion (3,740, 3.94%), GI hemorrhage (1,210, 1.28%), and hematoma (1,190, 1.25%) were most common. Fluoxetine demonstrated the highest proportion of hemorrhage (unspecified) among all SSRIs. CNS hemorrhages, including cerebral hemorrhage (375, 0.40%) and subarachnoid hemorrhage (336, 0.35%), were consistently reported. Additional bleeding events included hematuria (340, 0.36%), rectal hemorrhage (294, 0.31%), purpura (285, 0.30%), and ecchymosis (282, 0.30%).

Although limited to 7,816 cases, fluvoxamine demonstrated a distinct bleeding profile. The most frequently reported events were hemorrhage (unspecified) (468, 5.99%), hematoma (100, 1.28%), gastritis hemorrhage (80, 1.02%), and hematemesis (77, 0.99%). Notably, GI and oral cavity bleeding events showed patterns not prominently observed with other SSRIs, including gingival bleeding (54, 0.69%) and mouth hemorrhage (48, 0.61%). Additional events included GI hemorrhage (66, 0.84%), contusion (55, 0.70%), epistaxis (44, 0.56%), and retinal hemorrhage (38, 0.49%).

Sertraline, with 108,307 cases representing the highest absolute number of reports, showed hemorrhage (unspecified) (11,778, 10.87%), contusion (4,400, 4.06%), hematoma (1,510, 1.39%), and epistaxis (748, 0.69%) as the most frequent bleeding events. CNS hemorrhages were notable findings, including subdural hematoma (620, 0.57%) and intracranial hemorrhage (570, 0.53%). Respiratory hemorrhages, including hemoptysis (455, 0.42%) and pulmonary alveolar hemorrhage (440, 0.41%), as well as hematuria (400, 0.37%) and GI hemorrhage (396, 0.37%), were also consistently reported (Table 5, Figure 4).

## Discussion

This large-scale pharmacovigilance study provides a comprehensive real-world characterization of hemorrhagic adverse event profiles associated with six frequently prescribed SSRIs, using data from the FAERS. By analyzing over 380,000 total SSRI reports and 5,604 bleeding-related cases, this study offers one of the most extensive descriptive evaluations of SSRI-associated bleeding patterns to date. In line with the study objectives, the findings support the established concept that bleeding complications represent a class effect of SSRIs, while also demonstrating observable differences in reporting patterns in bleeding phenotype and anatomical distribution.

GI bleeding emerged as the most frequently reported category of bleeding across all SSRIs evaluated. Unspecified GI hemorrhage was consistently observed for all six drugs (ranging from 0.37% with sertraline to 1.45% with citalopram), alongside rectal hemorrhage (0.31%-5.94%), hematochezia (0.76%-2.84%), and hematemesis (0.22%-0.98%). Escitalopram demonstrated particularly high proportions of rectal hemorrhage (2,562, 5.94%) and hematochezia (1,224, 2.84%), while citalopram (405, 0.76%) and paroxetine (notable but lower frequencies) also reported hematochezia among leading GI events. These observations are concordant with epidemiological studies and meta-analyses reporting an increased risk of GI bleeding among SSRI users [3,10]. Mechanistically, these findings are biologically plausible. Inhibition of the platelet serotonin transporter leads to depletion of intraplatelet serotonin stores, impairing platelet aggregation at sites of vascular injury [4-6]. Additionally, SSRIs may enhance gastric acid secretion through vagal stimulation, potentially compromising mucosal integrity and predisposing to upper GI bleeding. The combination of impaired platelet-mediated hemostasis and increased GI mucosal vulnerability provides a coherent explanation for the predominance of GI bleeding events observed across the SSRI class.

The relatively high frequency of rectal hemorrhage reported for escitalopram should be interpreted with caution. This finding may reflect reporting artefacts, including stimulated reporting, variations in coding practices, or clustering of related PT within MedDRA, rather than a true pharmacological difference. In addition, confounding factors such as concomitant medications and underlying GI disease cannot be excluded in FAERS data. Therefore, this observation should be considered hypothesis-generating and requires confirmation in controlled epidemiological studies.

Frequent reports of contusions and hematomas across all SSRIs represent another important finding, though their interpretation warrants nuance. Contusions and superficial mucocutaneous bleeding events (such as epistaxis and gingival bleeding) are classically associated with disorders of primary hemostasis, whereas hematomas are more often linked to abnormalities of secondary hemostasis. In this analysis, contusions were consistently among the most frequently reported events (ranging from 0.70% with fluvoxamine to 10.24% with citalopram), while hematomas were also commonly reported (1.25%-4.52% across most SSRIs). This mixed pattern suggests that SSRI-associated bleeding may not represent a pure primary hemostatic disorder but rather a spectrum of bleeding manifestations predominantly driven by platelet dysfunction, with potential modulation by patient-specific factors and concomitant therapies. The prominence of superficial bleeding manifestations nonetheless remains consistent with the established mechanism of SSRI-induced impairment of platelet aggregation via serotonin depletion [4-6].

Although CNS hemorrhages were reported less frequently than GI or mucocutaneous events, their occurrence across multiple SSRIs is clinically important given their potential severity. Cerebral hemorrhage (0.40%-1.27%), subarachnoid hemorrhage (0.35%-1.16%), subdural hematoma, and intracranial hemorrhage were documented for several agents. Citalopram demonstrated the highest proportions of CNS bleeding events, including cerebral hemorrhage (675, 1.27%) and subarachnoid hemorrhage (616, 1.16%), followed by paroxetine and fluoxetine. These findings align with prior observational studies that have reported inconsistent but concerning associations between SSRI use and intracranial hemorrhage, particularly in elderly patients and those receiving concomitant antithrombotic therapy [10,11]. The present analysis reinforces the need for heightened clinical vigilance when prescribing SSRIs to patients with elevated baseline cerebrovascular risk.

Distinct drug-specific bleeding patterns were also observed. However, these apparent differences should be interpreted with caution, as FAERS data cannot reliably distinguish true biological differences from variations in reporting behavior, prescribing practices, geographic distribution, or regional coding patterns. As such, these findings reflect reporting tendencies rather than definitive pharmacological phenotypes. However, these differences should be interpreted with caution, as FAERS data cannot confirm true biological differences between individual SSRIs. Escitalopram showed distribution characterized by markedly elevated rates of rectal hemorrhage (2,562, 5.94%) and hematochezia (1,224, 2.84%), in addition to prominent respiratory tract bleeding events such as hemoptysis (535, 1.24%) and pulmonary alveolar hemorrhage (495, 1.15%). This constellation may suggest possible anatomical site predilections; however, differential reporting behavior, indication patterns, and case volume differences cannot be excluded and warrant cautious interpretation. Fluvoxamine, despite a smaller number of reported cases, demonstrated a bleeding profile dominated by hemorrhagic gastritis (80, 1.02%), hematemesis (77, 0.99%), gingival bleeding (54, 0.69%), mouth hemorrhage (48, 0.61%), and retinal hemorrhage (38, 0.49%). One potential explanation may relate to fluvoxamine’s potent inhibition of cytochrome P450 enzymes, which could increase exposure to concomitant medications known to impair gastric mucosal integrity or hemostasis, such as NSAIDs (e.g., ibuprofen, diclofenac) or antiplatelet agents (e.g., aspirin, clopidogrel) [8,9]. However, this hypothesis remains highly speculative and cannot be confirmed within the constraints of spontaneous reporting data. The FAERS dataset does not allow assessment of pharmacokinetic interactions, temporal relationships, or medication exposure levels, and therefore, this interpretation should be considered hypothesis-generating rather than explanatory. Importantly, citalopram and escitalopram agents with minimal CYP450 inhibition also demonstrated substantial bleeding event rates, supporting the concept that platelet serotonin depletion alone may be sufficient to confer clinically meaningful bleeding risk independent of metabolic drug-drug interactions. These findings are consistent with prior literature indicating that no SSRI is entirely free from bleeding risk [3,11]. 

A clinically relevant observation from this study is that 541 (9.7%) of all reported SSRI-associated bleeding cases involved concomitant use of at least one medication known to increase bleeding risk. Antiplatelet agents were the ones most frequently co-reported (246, 4.4%), followed by NSAIDs (143, 2.6%), DOACs (130, 2.3%), and warfarin (22, 0.4%). Co-prescription rates varied across SSRIs, ranging from 3 (3.7%) with fluvoxamine to over 118 (11.5%) with citalopram and escitalopram. These findings are consistent with previous studies demonstrating synergistic or multiplicative bleeding risk when SSRIs are combined with NSAIDs or antiplatelet agents [7,10]. Given the frequent coexistence of depression with cardiovascular disease and chronic pain conditions, such combinations are often unavoidable. Nonetheless, these data underscore the importance of careful medication reconciliation, individualized risk-benefit assessment, and consideration of gastroprotective strategies or alternative therapies in patients receiving SSRIs alongside other bleeding-risk medications.

An additional consideration is the potential for indication bias. Patients prescribed SSRIs, particularly older adults or those with comorbid cardiovascular conditions, may have a higher baseline risk of bleeding independent of SSRI exposure. These populations are also more likely to receive concomitant therapies such as antiplatelet agents, anticoagulants, or NSAIDs, which further increase bleeding risk. Consequently, the observed bleeding events may reflect, in part, underlying patient characteristics and treatment patterns rather than the pharmacological effects of SSRIs alone. This limitation is inherent to observational pharmacovigilance data and should be considered when interpreting these findings.

Future research should integrate spontaneous reporting data with population-based cohort studies to quantify absolute and relative bleeding risks, evaluate dose-response relationships, and examine outcomes in vulnerable populations such as older adults. Comparative safety analyses across antidepressant classes may further inform safer prescribing strategies and individualized treatment decisions.

This study has several notable strengths. First, it represents one of the largest cumulative descriptive analyses of SSRI-associated hemorrhagic adverse events using a real-world pharmacovigilance database, encompassing 5,604 bleeding-related cases across six commonly prescribed SSRIs. Second, the use of standardized MedDRA terminology at the SOC and high-level group term levels ensured reproducible and systematic identification of hemorrhagic events. Third, the comparative evaluation across individual SSRIs allowed identification of both class-wide patterns and drug-specific bleeding phenotypes, providing clinically meaningful insights beyond aggregate SSRI analyses. Finally, the inclusion of concomitant bleeding-risk medication assessment enhances the clinical applicability of the findings by contextualizing bleeding events within real-world polypharmacy scenarios.

Limitations

This study has several limitations inherent to analyses based on spontaneous reporting systems. FAERS data are subject to underreporting, selective reporting, and reporting bias, which may overrepresent severe or clinically recognized bleeding events while underestimating milder manifestations. The absence of denominator data precludes estimation of incidence rates or relative risks, limits causal inference, and prevents calculation of exposure-adjusted reporting rates.

Clinical details, such as SSRI dose, duration of therapy, indication, laboratory parameters, and bleeding severity, were not consistently available. Additionally, reported bleeding events were not independently validated, and diagnostic accuracy cannot be confirmed. The database also does not allow a reliable determination of the temporal relationship between SSRI exposure and bleeding onset, further limiting causal interpretation.

A notable limitation of this analysis is the high proportion of missing age data, which ranged from approximately 40% to over 50% across SSRIs. This substantially limits the interpretation of age-specific findings and may introduce bias in the characterization of demographic patterns. Consequently, conclusions regarding age-related risk distributions should be interpreted with caution.

A substantial proportion of reports lacked complete demographic information, particularly age and geographic origin, which may affect subgroup interpretation. Confounding by comorbid conditions and unmeasured concomitant medications cannot be entirely excluded, and adjustment for these factors was not possible within this dataset. Furthermore, the potential for indication bias should be considered, as patients receiving SSRIs, particularly older adults and those with cardiovascular comorbidities, may have a higher baseline risk of bleeding and are more likely to receive concomitant antithrombotic therapies.

Although FDA-recommended deduplication methods were applied, the possibility of duplicate or incomplete reporting cannot be entirely excluded. In addition, this study was designed as a descriptive analysis, and formal disproportionality or signal detection analyses were intentionally not performed.

Despite these limitations, the large sample size and real-world nature of the dataset provide valuable insights into bleeding patterns associated with SSRI use in routine clinical practice. Nevertheless, the use of a large, publicly accessible pharmacovigilance database with standardized adverse event coding provides an important signal-detection and pattern-recognition framework that complements findings from controlled clinical trials and observational cohort studies.

Additional limitations should be noted. Confounding by comorbidities cannot be excluded, as underlying conditions may independently increase bleeding risk. Indication bias is also possible, as SSRIs are prescribed for diverse conditions with varying baseline risks. Furthermore, FAERS data are subject to duplicate reporting and missing information (e.g., demographics and concomitant medications), which may affect accuracy. Therefore, findings should be considered descriptive and hypothesis-generating rather than causal. Use of a predefined MedDRA HLGT may have excluded certain bleeding events coded outside this hierarchy. Additionally, MedDRA versions were not standardized across the study period; however, no significant impact on overall findings was expected.

## Conclusions

This large FAERS-based pharmacovigilance analysis demonstrates that bleeding represents a class-wide safety concern among SSRIs, with variability in reported bleeding patterns within a descriptive context. However, these findings are descriptive and do not imply causal relationships or comparative safety differences between individual SSRIs. GI bleeding was the most frequently reported category, followed by mucocutaneous events and, less commonly, CNS hemorrhages. Concomitant use of bleeding-risk medications in 9.7% of cases highlights the clinical importance of polypharmacy. These findings underscore the need for careful medication reconciliation, individualized risk assessment, and heightened clinical vigilance, particularly in older adults and patients receiving antithrombotic therapy. Further studies integrating pharmacovigilance and population-based data are needed to better quantify bleeding risk and inform safer prescribing.
